# Comparison of cerebrovascular reactivity recovery following high‐intensity interval training and moderate‐intensity continuous training

**DOI:** 10.14814/phy2.14467

**Published:** 2020-06-07

**Authors:** Joel S. Burma, Alannah Macaulay, Paige Copeland, Omeet Khatra, Kevin J. Bouliane, Jonathan D. Smirl

**Affiliations:** ^1^ Sport Concussion Research Lab University of British Columbia Kelowna BC Canada; ^2^ Sport Injury Prevention Research Centre Faculty of Kinesiology University of Calgary Calgary AB Canada; ^3^ Hotchkiss Brain Institute University of Calgary Calgary AB Canada; ^4^ Human Performance Laboratory University of Calgary Calgary AB Canada; ^5^ Integrated Concussion Research Program University of Calgary Calgary AB Canada; ^6^ Faculty of Medicine University of British Columbia Vancouver BC Canada; ^7^ Alberta Children's Hospital Research Institute University of Calgary Calgary AB Canada; ^8^ Libin Cardiovascular Institute University of Calgary Calgary AB Canada

**Keywords:** acute recovery, cerebral blood flow, cerebrovascular reactivity, exercise, hyperventilation, rebreathing

## Abstract

A common inclusion criterion when assessing cerebrovascular (CVR) metrics is for individuals to abstain from exercise for 12–24 hr prior to data collections. While several studies have examined CVR *during* exercise, the literature describing CVR throughout post‐exercise recovery is sparse. The current investigation examined CVR measurements in nine participants (seven male) before and for 8 hr following three conditions: 45‐min moderate‐continuous exercise (at ~50% heart‐rate reserve), 25‐min high‐intensity intervals (ten, one‐minute intervals at ~85% heart‐rate reserve), and a control day (30‐min quiet rest). The hypercapnic (40–60 mmHg) and hypocapnic (25–40 mmHg) slopes were assessed via a modified rebreathing technique and controlled stepwise hyperventilation, respectively. All testing was initiated at 8:00a.m. with transcranial Doppler ultrasound measurements to index cerebral blood velocity performed prior to the condition (pre) with serial follow‐ups at zero, one, two, four, six, and eight hours within the middle and posterior cerebral artery (MCA, PCA). Absolute and relative MCA and PCA hypercapnic slopes were attenuated following high‐intensity intervals at hours zero and one (*all p < *.02). No alterations were observed in either hypocapnic or hypercapnic slopes following the control or moderate‐continuous exercise (*all p > *.13), aside from a reduced relative hypercapnic MCA slope at hours zero and one following moderate‐continuous exercise (*all p < *.005). The current findings indicate the common inclusion criteria of a 12–24 hr time restriction on exercise can be reduced to two hours when performing CVR measures. Furthermore, the consistent nature of the CVR indices throughout the control day indicate reproducible testing sessions can be made between 8:00a.m. and 7:00p.m.

## Graphical abstract

Absolute and relative MCA and PCA cerebrovascular reactivity hypercapnic slopes were attenuated following high‐intensity intervals at hours zero and one (all *p* < .02). No alterations were observed in either hypocapnic or hypercapnic slopes following the control or moderate‐continuous exercise (all *p* > .13), aside from a reduced relative hypercapnic MCA slope at hours zero and one following moderate‐continuous exercise (all *p* < .005). The current findings indicate the common inclusion criteria of a 12–24 hr time restriction on exercise can be reduced to two hours when performing CVR measures.

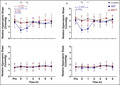

## 1 INTRODUCTION

The cerebrovasculature is highly sensitive to alterations in carbon dioxide as vasodilation and vasoconstriction occurs during hypercapnic and hypocapnic stimuli, respectively (Fierstra et al., 2013; Kety & Schmidt, 1945). The extent of change in cerebral blood flow velocity (CBV) for a given stimulus is termed cerebrovascular reactivity (CVR) and is thought to be an indication of vascular reserve and autoregulatory efficiency within the brain (Ito, Kanno, Ibaraki, Hatazawa, & Miura, 2003; Nur et al., 2009). The changes to CBV can be indexed in real‐time with transcranial Doppler ultrasound (TCD) (Willie et al., 2011). One of the most common methods to assess reactivity to hypercapnic stimuli is the rebreathing method, in which participants breathe into a closed system and inhale part or all the gases previously exhaled thereby reducing the alveolar‐arterial difference in carbon dioxide levels (Read, 1967). As the test proceeds, the participant will consume the excess oxygen present and in turn produce carbon dioxide as a metabolic by‐product, with the resulting hypercapnic levels leading to an increase in CBV via cerebrovasodilation (Fierstra et al., 2013; Poulin & Robbins,). Conversely, the alterations with hypocapnia are typically assessed through controlled hyperventilation, in which participants increase their tidal volume and breathing frequency to reduce the partial pressure of end‐tidal volume of carbon dioxide (P_ET_CO_2_) in a stepwise manner (Krainik, Hund‐Georgiadis, Zysset, & Von Cramon, 2005; Mäkiranta et al., 2004; Posse, Weckesser, Müller‐Gärtner, & Dager, 1996; Weckesser et al., 1999). This leads to vasoconstriction of the cerebrovasculature ultimately causing a reduction in CBV (Posse et al., 1996). Furthermore, by combining the hypercapnic response with rebreathing and the hypocapnic response with controlled hyperventilation researchers have been able to accurately describe the real‐time sigmoidal response to CO_2_ when coupled with TCD (Battisti‐Charbonney, Fisher, & Duffin, 2011; Claassen, Zhang, Fu, Witkowski, & Levine, 2007).

To date there have been a very limited number of studies which have investigated how exercise impacts CVR. Rasmussen and colleagues (2006)—demonstrated exercise resulted in an elevated response of CBV to a given change in P_ET_CO_2_, which was further increased during more strenuous exercise. Similarly, these results are comparable to other studies which have found the hypercapnic slope was augmented during exercise to exhaustion (Ogoh, Ainslie, & Miyamoto, 2009; Ogoh, Hayashi, Inagaki, Ainslie, & Miyamoto, 2008). A final study conducted examining CVR over the course of a 12‐week training program found CVR to hypercapnia was elevated during exercise at both 30 and 70% of heart rate reserve compared measures before the training program, irrespective of age (Murrell et al., 2013). While all these previous studies have examined the CVR response *during exercise*, to our knowledge there have been no studies conducted examining how CVR is altered *during acute recovery* following exercise. Furthermore, there is an absence of literature investigating CVR with high intensity intervals as the previous studies described used either steady‐state or incremental‐exercise.

Currently, inclusion criteria for research investigations which have examined CVR parameters required participants to abstain from exercise for 12 (Ainslie, Hamlin, Hellemans, Rasmussen, & Ogoh, 2008; Battisti‐Charbonney et al., 2011; Claassen et al., 2007; Lucas et al., 2010; Peebles, Ball, MacRae, Horsman, & Tzeng, 2012; Regan, Fisher, & Duffin, 2014; Skow et al., 2013; Strohm, Duffin, & Fisher, 2014) or 24 hr (Ainslie et al., 2007; Cummings, Swart, & Ainslie, 2007; Ogoh et al., 2008, 2009; Willie et al., 2012). Despite the many studies employing these guidelines, to our knowledge there have been no studies which have objectively quantified the duration for which CVR is impacted throughout recovery period following an acute bout of maximal or submaximal exercise. Therefore, the purpose of this study was to determine the duration an acute bout of exercise impacts CVR, and the extent to which this is dependent on exercise intensity. To address this aim, the exercise modalities employed in the current investigation were: moderate intensity continuous training (MICT); high intensity interval training (HIIT) and a control rest day (to determine the within‐day reproducibility of CVR responses). These two exercise intensities were chosen to challenge the phenomena of hypercapnic‐induced vasodilation and hyperventilation‐induced vasoconstriction following MICT and HIIT, respectively. It was hypothesized CBV would be increased throughout the CVR assessment immediately following the MICT condition, whereas it would be decreased following the HIIT protocol compared to their own respective baseline values. Furthermore, it was hypothesized the hypercapnic and hypocapnic slopes would be greater following the MICT condition due to the vessel having a greater ability to maximally dilate, therefore having a higher CBV at eucapnia. Conversely, the slopes would be attenuated following the HIIT protocol, as the induced vasoconstriction would impede the ability of the cerebrovasculature to maximally dilate at eucapnia, leading to reduced CBV. These changes would be present immediately following the exercise conditions and would return to baseline as the recovery duration progressed.

## 2 METHODS

### 2.1 Study design and participants

**FIGURE 1:**
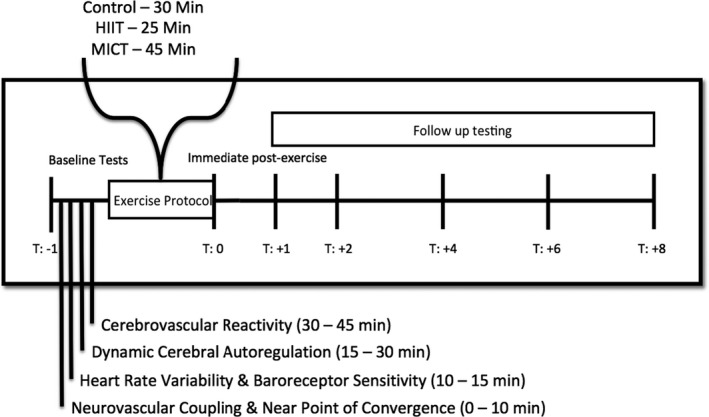
Testing order of the cardiovascular and cerebrovascular measures performed at each time point across the day as previously described (Burma et al., 2020). Baseline measures were collected each day before engaging in a randomly selected exercise condition. Each serial follow‐up measure post‐exercise had a 15‐min washout period to ensure the cerebrovascular reactivity did not influence the subsequent measures. Testing was initiated at 8:00 a.m. and was fully completed by 7:00 p.m. each testing day

Using a randomized cohort design, seven males and two females (*n* = 9) and young adults (26 ± 5 years and 25 ± 4 kg/cm^2^) were recruited from the university setting to partake in this study. Participants came in on three separate days (control, HIIT, and MICT), which was separated by a minimum of at least three days and had an average of 22 ± 17 days between interventions and took an average of 43 ± 28 days to complete all three testing sessions. None of the participants were excluded based upon any history of neurological, cerebrovascular, cardiorespiratory, or musculoskeletal complications and all participants had moderate to good fitness levels. Both the hypercapnic and hypocapnic protocols were collected at baseline prior to each randomized condition and again during follow up at hours zero, one, two, four, six, and eight (Figure 1). Testing commenced at the same time each day (8:00 a.m.) to limit any potential impact diurnal variation may contribute to the results (Conroy, Spielman, & Scott, 2005). To limit any effect the variation of hormones may have on CBV during the menstrual cycle, female participants were tested between days 3 and 7 of the early follicular phase, as reproductive hormones are thought to be relatively stable during this time period (Boivin & Shechter, 2010). Due to the small number of females recruited for the current study (and control employed about the menstrual cycle), it is not possible to determine any potential effects of sex differences, as such all participants were included in the final analyses. All testing protocols were demonstrated and thoroughly explained to each participant prior to testing, ensuring they were familiar and confident to perform the procedures. Participants withdrew from exercise, caffeine, and alcohol 12 hr before engagement in this investigation, to be consistent with previous studies examining CVR metrics during exercise (Ainslie et al., 2008; Battisti‐Charbonney et al., 2011; Claassen et al., 2007; Lucas et al., 2011; Peebles et al., 2012; Regan et al., 2014; Skow et al., 2013; Strohm et al., 2014). Additionally, dietary factors across the day of testing were controlled for by having participants consume two meal replacement drinks (Vanilla Nutrition Shake, Kirkland Signature; 210 calories each) immediately following the two hour post exercise follow up time point. Finally, participants had access to water, two sport drinks (Gatorade Perform, PepsiCo; 150 calories each), and were allowed to use the washroom as needed.

This was a subsample of a larger investigation examining the effect different exercise intensities (MICT and HIIT) have on cardiovascular (cardiac baroreceptor sensitivity and heart rate variability) and cerebrovascular function (neurovascular coupling, cerebrovascular reactivity, and dynamic cerebral autoregulation (Burma et al., 2020)). To ensure interpretability of the results between and within conditions, each cardiovascular and cerebrovascular measure was collected in the same order at each time point (Figure 1). This aspect of the overall experiment examines how CVR measures are impacted following MICT and HIIT exercise, as there is currently no literature describing either the effects of HIIT on CVR measures nor the recovery trajectory of these measures. Participants provided written informed consent before participating and the study was approved by the University of British Columbia clinical ethics review board (H16‐00507).

### 2.2 Instrumentation

Heart rate was assessed using a three‐lead electrocardiogram. The right middle cerebral artery (MCA) and left posterior cerebral artery (PCA) blood velocities were insonated via TCD (Spencer Technologies). Two 2‐Mhz ultrasound probes (Spencer Technologies) were placed over the right and left temporal acoustic windows to measure CBV. Once vessels were identified, the ultrasound probes were locked into place using a fitted head frame and confirmed using carotid artery compressions and a visual task (Willie et al., 2011). Beat‐to‐beat blood pressure was recorded using finger photoplethysmography with a brachial cuff to correct for the difference in hydrostatic pressure gradient between finger and brachial artery heights (Finometer PRO, Finapres Medical Systems). During all CVR assessments and exercise bouts, participants were instructed to have space between their finger with the photoplethysmography and their other fingers. They were also instructed to not squeeze or press the finger against the bike handles to ensure an adequate mean arterial pressure trace was collected. Furthermore, P_ET_CO_2_ was measured with an online gas analyzer (ML206, ADInstruments) and was calibrated with known gas concentrations prior‐to data collections. Data were recorded at 1,000 Hz (PowerLab 8/30 ML880, ADInstruments), time locked and stored for offline analysis with commercially available software (LabChart version 7.1, ADInstruments).

### 2.3 Experimental protocols

**FIGURE 2:**
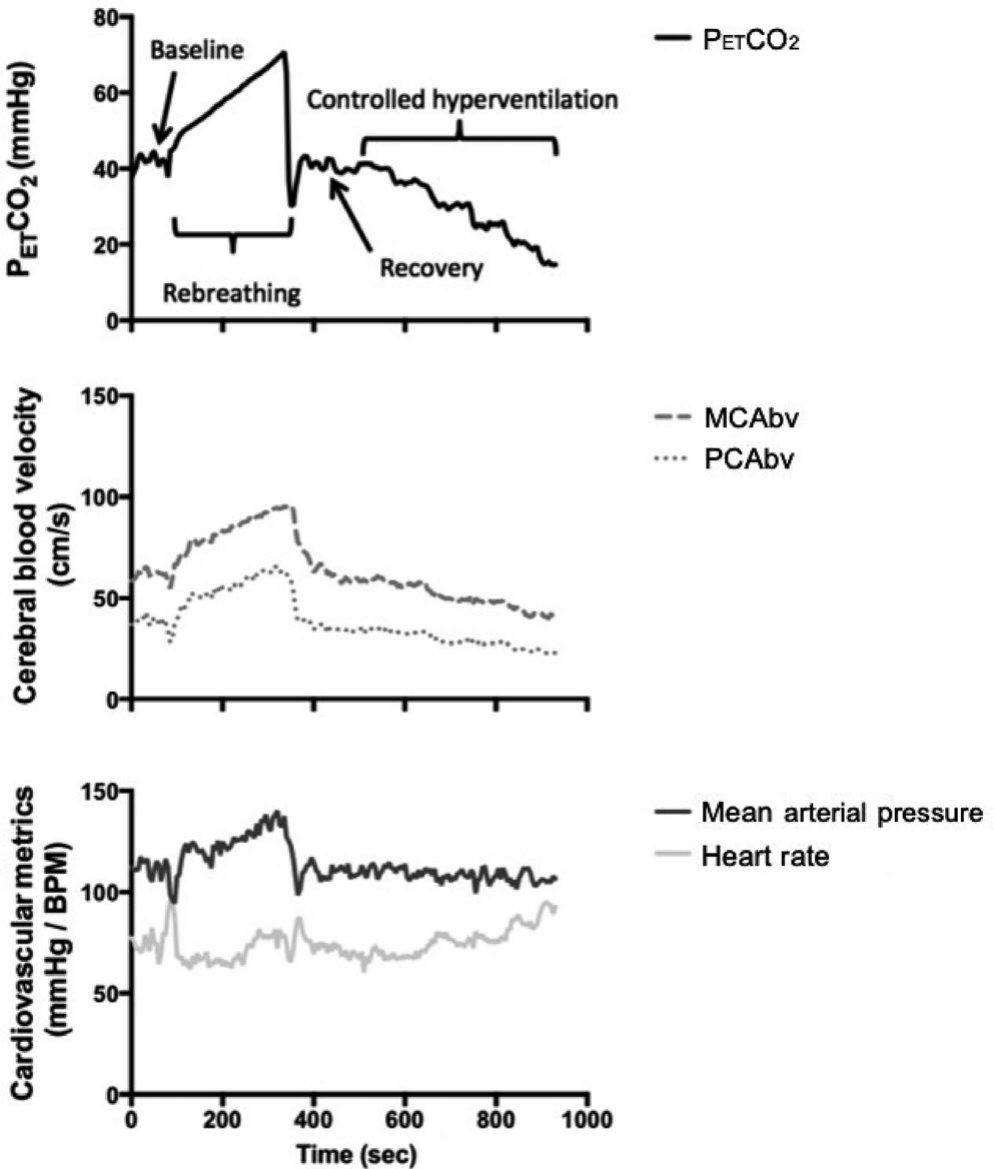
Representative CVR trace from a single participant demonstrating the hypercapnic rebreathing protocol (40–70 mmHg) and the hypocapnic step‐down controlled hyperventilation 15–40 mmHg). End tidal values of carbon dioxide (P_ET_CO_2_), middle cerebral artery blood velocity (MCAbv), posterior cerebral artery blood velocity (PCAbv), mean arterial pressure, and heart rate traces are shown. Note: the reduction in CBV at the completion of baseline and in the initial 15 s of the rebreathing protocol was due to the instructed over breathing of the subject by the investigator to equilibrate their breathing with the circuit (Skow et al., 2013)

Both of the hypercapnic and hypocapnic states were assessed with TCD in the PCA and MCA as this has been demonstrated to be a valid measure of CVR (Ainslie & Duffin, 2009; Fierstra et al., 2013). Hypercapnia was assessed via a closed circuit rebreathing bag filled with 93% oxygen and 7% carbon dioxide, whereas hypocapnia was assessed through controlled stepwise hyperventilation. For the former, participants were seated upright (McDonnell et al., 2013) and breathed at their normal respiration rate (P_ET_CO_2_ = ~40 mmHg) for one to two minutes to acquire baseline values on an open circuit prior to initiating the rebreathing protocol. At the end of the baseline period, participants performed three maximal ventilations before the breathing apparatus was switched from an open to closed circuit (Ainslie & Duffin, 2009; Read, 1967). When the transition from room air to rebreathing protocol occurred, the participants maximally ventilated for the first three breathes to equilibrate the rebreathing circuit (Skow et al., 2013), after which they were instructed to breathe normally until they were no longer able to continue or their P_ET_CO_2_ reached a value of 70 mmHg (Read, 1967). The circuit was then opened to room air and the participants were given three‐to‐five minutes to recover. Following this, hypocapnia was then assessed through a controlled stepwise hyperventilation protocol, as previously mentioned. For this purpose, measures for P_ET_CO_2_ once again started at ~40mmHg and controlled hyperventilation was employed to elicit 5 mmHg reductions in a stepwise fashion to a final level of 15 mmHg. Each stage (40, 35, 30, 25, and 20 mmHg) was held for 60 s to minimize any potential effects transit time (~20 s) may have on CBV. Therefore, a conservative approach was taken with the final 20 s of each increment used for analysis. Figure 2 shows a representative trace from the data collections highlighting the effects of the hypercapnic and hypocapnic stimuli on the physiologically relevant markers.

### 2.4 Exercise protocols

Participants completed the exercise conditions (HIIT and MICT) on a cycle ergometer (ergoline GmbH, Lindenstr, Germany) and a control day during which they sat quietly for 30 min. The HIIT protocol lasted 25 min and was comprised of warmup, exercise, and cool down phases, broken down as follows. Participants were given three minutes to warm up before performing ten one‐minute intervals of exercise at 85%–90% heart rate reserve (HRR).

$$\begin{array}{cc} \text{HRR} & =\text{Target intensity}\left(\text{Age}\;-\;\text{Predicted Maximum Heart Rate}\;-\;\text{Resting Heart Rate}\right)\\ & \;+\text{Resting Heart Rate} \end{array}$$

Between each minute of work, participants cycled at roughly 15% of the power output (Jung, Bourne, Beauchamp, Robinson, & Little, 2015). Three minutes of cool down was allotted after the final interval to transition the subject for the follow‐up measures before starting testing at time zero. The MICT condition consisted of 45 min of exercise at a 50%–60% HRR, with a five minute warm‐up protocol (Wewege, van den Berg, Ward, & Keech, 2017). The two exercise modalities were selected because they represent stimuli which differentially impact CBV (Ogoh & Ainslie, 2009a; Ogoh et al., 2007; Smirl, Haykowsky, Nelson, Altamirano‐Diaz, & Ainslie, 2012). The maximal elevation in CBV occurs during exercise at ~60%–70% of VO_2max_, which was the estimated workload of the MICT condition; (Marsden et al., 2012; Ogoh & Ainslie, 2009b) whereas, the HIIT workload was 85%–90% of heart rate reserve, as this workload provides a stimulus in which CBV would be nearing a return to baseline levels due to hyperventilation‐induced cerebral vasoconstriction (Marsden et al., 2012; Ogoh & Ainslie, 2009b; Ogoh et al., 2005). While the exercise modalities are being performed in a controlled laboratory environment, they were also selected as being broadly representative of real‐world sporting environments. The MICT protocol is similar to events such as distance running or cycling races, which require athletes to engage in longer, steady state exercise. Contrarily, the HIIT protocol requires individuals to perform short‐bouts of maximal exertion, which are more representative of a hockey or football environment.

### 2.5 Data processing

Beat‐to‐beat blood pressure and CBV were calculated using diastolic and systolic traces from the R‐R intervals collected by the electrocardiogram. P_ET_CO_2_ levels were measured by examining breath‐to‐breath peak expired carbon dioxide levels with all signals being visually inspected for artefacts. The blood velocities of the MCA and PCA at P_ET_CO_2_ values of 20, 25, 30, 35, and 40 mmHg over the final 20 s of each stage during the controlled stepwise hyperventilation were used for analysis. Additionally, the MCA and PCA blood velocities during the modified rebreathing technique were taken with an average of 20 s around P_ET_CO_2_ values of 40, 50, 55, and 60 mmHg. Furthermore, the relative and absolute CVR analysis was only performed on the data associated with typical physiological range of MCA and PCA (P_ET_CO_2_ range of 25–60 mmHg; Hypocapnic: 25–40 mmHg; hypercapnic: 40–60 mmHg) (Ainslie et al., 2008; Claassen et al., 2007). The additional hypocapnic stages below 25 mmHg and hypercapnic rebreathing performed above 60 mmHg were performed to ensure maximal dilation and constriction of the pial vessels had occurred. Finally, the critical closing pressure (CrCP) was determined within both the MCA and the PCA, using a simplified regression technique (Panerai, 2003). In brief, this was done by plotting CBV as a function of arterial blood pressure (i.e., systolic and diastolic), determine the regression slope of all the data points, and calculating when the slope crossed the x‐axis, which was deemed the approximate CrCP (Panerai, 2003). The CBV and arterial blood pressure values were calculated over ten seconds of data at P_ET_CO_2_ values of 25, 40, and 55mmHg at each time point and during the three interventions.

### 2.6 Statistical analyses

Potential differences across exercise conditions and time were analysed using a three (condition: HIIT, MICT, control) by seven (time: baseline, zero, one, two, four, six, and eight hour follow ups) Repeated Measures ANOVA implemented in SPSS version 25.0. Bonferroni post‐hoc analyses were conducted to determine significant condition effects. A priori Bonferroni corrected simple effects comparisons were performed between the follow up time points and the baseline data to establish when the CVR measures were no longer different from the baseline data. Data are presented as mean ± *SD*. Significance was set a priori at *p *<* *.05.

## 3 RESULTS

### 3.1 Cerebrovascular and cardiovascular parameters

At baseline, there were no differences in the MCA blood velocities, PCA blood velocities, or the P_ET_CO_2_ values in each condition (all *p* > .287), with these measures having a good coefficient of variation (CoV) reproducibility of <7%, <9%, and <5%, respectively (Table 1). Similarly, mean arterial pressure across the cardiac cycle and heart rate were stable at baseline measures in all three conditions (all *p* > .640), as indicated by the CoV reproducibility of <8% and <4%, respectively (Table 1).

**Table 1:** Cardiovascular and cerebrovascular variables during a one‐minute baseline prior to each CVR assessment at baseline, during, and following 30‐min of control rest, 45‐min of moderate‐intensity continuous training (MICT), and 25‐min of high‐intensity interval training (HIIT)

	Pre	During	T0	T1	T2	T4	T6	T8
Control								
P_ET_CO_2_ (mmHg)	39 ± 3	38 ± 2	39 ± 2	38 ± 3	38 ± 3	39 ± 3	39 ± 3	38 ± 4
MCAbv (cm/s)	60 ± 9	63 ± 7	61 ± 8	60 ± 8	61 ± 9	60 ± 10	62 ± 6	59 ± 10
MCA CrCP (mmHg)	12 ± 3	9 ± 2	11 ± 2	12 ± 2	11 ± 3	9 ± 2	11 ± 4	8 ± 3
PCAbv (cm/s)	37 ± 6	38 ± 6	38 ± 6	38 ± 6	38 ± 5	36 ± 7	36 ± 5	36 ± 6
PCA CrCP (mmHg)	16 ± 2	17 ± 4	16 ± 3	17 ± 1	14 ± 3	14 ± 2	13 ± 3	15 ± 4
MAP (mmHg)	93 ± 9	95 ± 10	94 ± 7	93 ± 7	91 ± 5	90 ± 5	92 ± 8	97 ± 4
HR (bpm)	66 ± 8	68 ± 8	64 ± 9	63 ± 12	63 ± 10	68 ± 12	65 ± 11	64 ± 11
HIIT								
P_ET_CO_2_ (mmHg)	40 ± 2	31 ± 4^*†^	37 ± 2^*^	38 ± 2	38 ± 2	40 ± 2	39 ± 2	39 ± 3
MCAbv (cm/s)	60 ± 9	58 ± 11	55 ± 11	55 ± 10	55 ± 10	58 ± 9	57 ± 7	57 ± 5
MCA CrCP (mmHg)	10 ± 1	3 ± 2^*†^	4 ± 1^*†^	5 ± 2^*†^	9 ± 2	8 ± 3	8 ± 2	9 ± 3
PCAbv (cm/s)	39 ± 8	36 ± 5	35 ± 7	35 ± 8	35 ± 8	38 ± 9	37 ± 7	38 ± 5
PCA CrCP (mmHg)	15 ± 3	5 ± 4^*†^	6 ± 2^*†^	13 ± 2	16 ± 4	13 ± 3	12 ± 2	16 ± 3
MAP (mmHg)	92 ± 9	113 ± 14^*†^	93 ± 10	95 ± 8	94 ± 11	92 ± 12	90 ± 9	95 ± 7
HR (bpm)	70 ± 11	167 ± 8^*†^	90 ± 11^*†^	81 ± 13^†^	74 ± 10	79 ± 9	73 ± 13	69 ± 12
MICT								
P_ET_CO_2_ (mmHg)	38 ± 3	40 ± 4^‡^	38 ± 2	39 ± 2	39 ± 1	40 ± 1	40 ± 2	39 ± 1
MCAbv (cm/s)	60 ± 10	69 ± 6^*†‡^	60 ± 9	61 ± 10	61 ± 10	64 ± 11	64 ± 8	62 ± 11
MCA CrCP (mmHg)	9 ± 2	5 ± 2^*†^	8 ± 3^‡^	9 ± 2	8 ± 1	10 ± 3	9 ± 3	8 ± 2
PCAbv (cm/s)	39 ± 7	43 ± 4^*†‡^	39 ± 6	39 ± 7	40 ± 6	41 ± 6	41 ± 7	41 ± 7
PCA CrCP (mmHg)	17 ± 2	13 ± 3^‡^	14 ± 2^‡^	17 ± 4	14 ± 3	13 ± 2	17 ± 4	12 ± 3
MAP (mmHg)	93 ± 7	109 ± 20	93 ± 10	94 ± 9	92 ± 7	94 ± 10	93 ± 7	92 ± 10
HR (bpm)	67 ± 9	136 ± 4^*†‡^	78 ± 16^†^	72 ± 16	68 ± 16	77 ± 15	69 ± 15	70 ± 15

Note

Values are means ± standard deviation. The asterisk (*) detonates a value that is different than its own respective Pre value at *p* < .05. The dagger (†) detonates a value that is different from the control condition at each respective time point at *p* < .05. The diesis (‡) detonates a value that is different from the HIIT condition at each respective time point at *p* < .05. End tidal values of carbon dioxide (P_ET_CO_2_), millimeters of mercury (mmHg), middle cerebral artery blood velocity (MCAbv), centimeters per second (cm/s), posterior cerebral artery blood velocity (PCAbv), mean arterial pressure (MAP), beats per minute (bpm), middle cerebral artery (MCA), Critical Closing Pressure (CrCP), and posterior cerebral artery (PCA).

During the three conditions, measures of P_ET_CO_2_ were similar between MICT and the control condition (*p* = .225) but was reduced during HIIT compared to control (*p* < .001) and MICT (*p* < .001) (Table 1). The MCA blood velocity during HIIT was similar to control (*p* = .252), but MCA blood velocity was elevated during MICT relative to both control and HIIT (all *p* < .047) (Table 1). Similarly, PCA blood velocity was enhanced with MICT compared to control and HIIT (all *p* < .036), however HIIT was proportionate to control (*p* = .580) (Table 1). Furthermore, relative to the control condition, both HIIT (*p* < .001) and MICT (*p* < .001) caused elevations in heart rate, with HIIT elevating heart rate higher than MICT (*p* < .001) (Table 1). However, mean arterial pressure was only elevated during HIIT (*p* = .004), but not MICT (*p* = .180), in comparison to the control condition (Table 1). Additionally, no differences were found in mean arterial pressure between HIIT and MICT (*p* = .738) (Table 1).

Following the HIIT interventions, P_ET_CO_2_ was reduced at hour zero (*p* = .032); whereas, heart rate was elevated (*p* = .004) (Table 1). Moreover, at hour zero, heart rate was elevated within the HIIT (*p* < .001) and MICT (*p* = .025) conditions compared to control at hour zero (Table 1). However, from hour one and onwards, no differences were found in any cardiovascular or cerebrovascular parameter succeeding HIIT (all *p* > .114) (Table 1). Likewise, all aforementioned metrics were comparable between CVR assessments during control and MICT interventions (Table 1). Finally, MCA CrCP was different compared to baseline, during the HIIT exercise and at hours zero and one following (all *p* < .002); however, PCA CrCP was different from baseline during exercise and at hour zero following HIIT (all *p* < .014) (Table 1). Moreover these values were also different from both control and MICT values at the same respective timepoints (all *p* < .037) (Table 1). However, from hour one onwards and from hour two onwards, no CrCP differences were found within the PCA and MCA, respectively (all *p* > .132) (Table 1). Within the MICT condition, only the CrCP within the MCA during the exercise bout was different from both baseline and during the control condition (all *p* < .046) (Table 1). Nonetheless, from hour zero and onwards during the MICT, CrCP remained stable within both vessels (all *p* > .293) (Table 1). Conversely, no differences were found within both the MCA and PCA CrCP across the control day (all *p* > .374) (Table 1).

### 3.2 Cerebrovascular reactivity variations within exercise conditions

**FIGURE 3:**
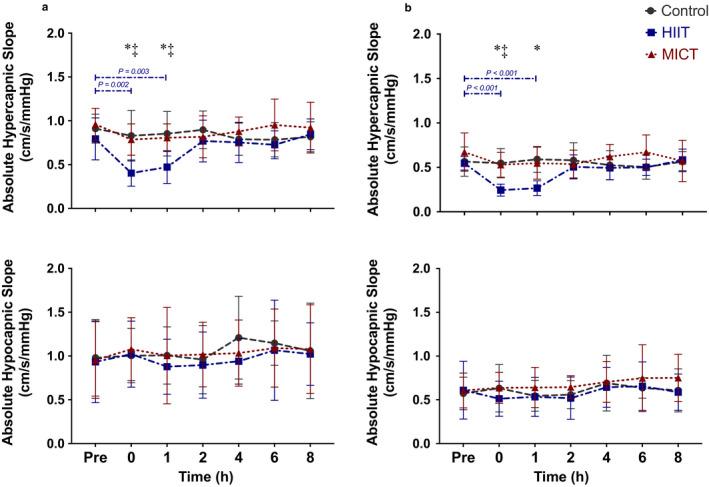
Absolute hypercapnic and hypocapnic slopes (mean ± standard deviation) across the day in the (a) middle cerebral artery and (b) posterior cerebral artery (*n* = 9). The asterisk (*) detonates a difference between control and high intensity interval training (HIIT) at hour zero (all *p* < .003) and hour one (all *p* < .004). The double cross (‡) detonates a difference between HIIT and moderate intensity continuous training (MICT) at time point zero (all *p* < .022) and one (all *p* = .005). The control condition is shown in grey (dashed), MICT in red (dotted), and HIIT in blue (dashed‐dotted)

**FIGURE 4:**
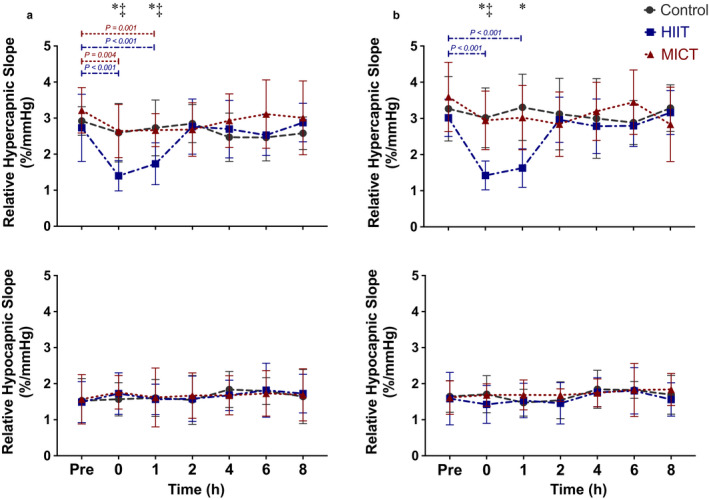
Relative hypercapnic and hypocapnic slopes across the day in the (a) middle cerebral artery and (b) posterior cerebral artery (*n* = 9). The values are displayed as mean ± standard deviation. The asterisk (*) detonates a difference between control and high intensity interval training (HIIT) at time point zero (all *p* < .003) and one (all *p* < .006). The double cross (‡) detonates a difference between HIIT and moderate intensity continuous training (MICT) at hours zero (all *p* < .027) and one (*p* = .010). The grey (dashed), red (dotted), and blue (dashed‐dotted) data points represent control, MICT, and HIIT, respectively

The relative and absolute MCA and PCA hypocapnic slopes were not different between conditions (all *p* > .310) (Figures 3 and 4). In contrast, the relative and absolute MCA and PCA hypercapnic slopes were decreased at least 37% (range: 37% – 55%) after HIIT exercise, which recovered by hour two (all *p* < .018) (Figures 3 and 4). Following MICT, the relative hypercapnic slope was decreased 20% at hour zero (*p* = .024) and 16% at hour one (*p* = .006) in the MCA (Figure 4a). However, all other MCA and PCA slopes did not differ from baseline on the MICT day (all *p* > .106) (Figures 3 and 4). Moreover all HIIT hypercapnic slopes were different than control and MICT at hour zero and one (all *p* < .022), excluding hour one between HIIT and MICT in the PCA (*p* = .081). However, there were no differences between control and MICT slopes at any time point in both vessels (all *p* > .170) (Figures 3 and 4). Moreover all relative and absolute slopes were the same across the control day (all *p* > .132) (Figures 3 and 4).

## 4 DISCUSSION

The current study was the first to examine the extent and duration MICT and HIIT modalities of exercise affect CVR during the post‐exercise recovery period. The key findings were: (a) both absolute and relative hypercapnic slopes were attenuated within the MCA and PCA for two hours following the HIIT protocol, but were unchanged following the MICT or control conditions, aside from a two hour reduction to the relative hypercapnic slope in the MCA following MICT; (b) there were no changes in absolute or relative hypocapnic slopes within the MCA or PCA across all three conditions; and (c) CVR measures were consistent across the control condition, indicting they had little variance using this methodology. Taken together, these results demonstrate the current inclusion criteria involving subjects to refrain from exercise for 12 to 24 hr prior to data collections examining CVR measures is unnecessarily conservative, as all CVR metrics returned to baseline levels two hours following high‐intensity exercise. Additionally, there was no indication of any within‐day variation in any CVR measures, as the control data CVR metrics were comparable across all seven time‐points within this current investigation.

### 4.1 Comparison with previous exercise studies

Several studies have examined how the CVR response is affected during various modalities of exercise (Ainslie et al., 2008; Ogoh et al., 2008, 2009; Rasmussen, Stie, Nielsen, & Nybo, 2006); however, this was the first study to examine CVR characteristics throughout an eight hour post‐exercise *recovery* period. Previous studies have demonstrated the hypercapnic CVR slope is augmented *during* both moderate (Murrell et al., 2013; Rasmussen et al., 2006) and exhaustive exercise (Fisher, Ogoh, Young, Raven, & Fadel, 2008; Ogoh et al., 2009), compared to control resting values. For example, Rasmussen and colleagues (2006) found this to be the case at a submaximal steady‐state exercise intensity of ~ 70% VO_2max_. This is similar to the results from this study as the hypercapnic relative slope was attenuated for one hour after exercise in the MCA, whereas no other slopes were affected follow moderate‐intensity exercise (~55%–60% HRR). Moreover the two investigations which examined the CVR response during incremental exercise intensity until exhaustion observed an increase in the hypercapnic curves (Ogoh et al., 2008, 2009). These studies had participants start at 20 Watts and increased 20 Watts every minute until they could no longer continue (Ogoh et al., 2008, 2009). Although the ultimate workload in these previous investigations would be a similar intensity to our HIIT condition, it differs as we had participants perform ten, one‐minute maximal bursts as opposed to two to three minutes at this intensity. The extended duration at high intensity in the current investigation may have contributed to a greater oxygen deficit being experienced (Gore & Withers, 1990). This could potentially explain some of the differences in results in the current investigation in which a prolonged two‐hour reduction in the hypercapnic slope occurred following HIIT exercise (Figures 3 and 4). Furthermore, the results of this investigation confirm the results of previous studies which have shown the hypocapnic curve is unaffected during exercise (Figures 3 and 4) (Ainslie & Duffin, 2009). Finally, in this study both the MCA and PCA responded in a homogeneous manner to both hypercapnia and hypocapnia during high intensity exercise. However, following the MICT protocol only the relative hypercapnic MCA slope was changed from baseline (Figure 4a). These results are consistent with a previous investigation, which also examined CVR across the entire relevant physiological range (~15 mmHg‐65 mmHg) (Willie et al., 2012). These authors found regional differences in reactivity within the brain where a greater reactivity was found within the vertebral artery compared to the internal carotid artery, MCA, and PCA (Willie et al., 2012). This finding demonstrates that although the extracranial neck arteries react differently to alterations in carbon dioxide, the intracranial vasculature responds in a global and uniform manner. However, this investigation found the intracranial vasculature to respond similarly to high‐intensity exercise, but differently to moderate exercise. The differences between the aforementioned study and this investigation is likely that this one measured CVR following exercise as opposed to at rest (Willie et al., 2012). Moreover, these authors noted the differences between regional cerebrovascular beds were potentially due to the posterior circulation being anatomically closer to the medulla oblongata, which is where the respiratory centre is located. As such, this area of the brain may be more sensitive to changes in carbon dioxide and therefore more heavily regulated, explaining the differences between the MCA and PCA following moderate exercise between circulations.

### 4.2 Potential mechanisms underlying the alterations between exercise conditions

Exercising up to an intensity of ~60%–70% of maximal oxygen uptake can be maintained for extended periods of time as this workout intensity is below the typical anaerobic threshold. As such, the increased ventilation associated with these sub‐anaerobic threshold stimuli occurs in a state of hyperpnea, thus there is a relatively equal increase in both oxygen consumption and the associated metabolic production of carbon dioxide (Mateika & Duffin, 1995). In turn, the elevation of carbon dioxide in the bloodstream resulting from the by‐products of aerobic metabolism (as indexed with P_ET_CO_2_) is associated with a corresponding increase in CBV, as a result of vasodilation occurring in the cerebrovasculature (Marsden et al., 2012; Ogoh & Ainslie, 2009b). However, at intensities above this threshold, the anaerobic threshold is surpassed, and the body shifts to anaerobic glycolysis (Beaver, Wasserman, & Whipp, 1986). This in turn, produces an elevation of carbon dioxide in the blood stream to buffer against an increase in acidity, which elicits a hyperventilation response (Beaver et al., 1986). The augmented respiration rate associated with anaerobic exercise intensities results in large reductions in arterial carbon dioxide levels which in turn induces vasoconstriction in the cerebrovasculature (Marsden et al., 2012; Ogoh & Ainslie, 2009b; Ogoh et al., 2005). In the current investigation, there was a reduction in the hypercapnic slope at hours zero and one following the HIIT condition (Figures 3 and 4) which indicates individuals experienced a slight inability to completely dilate the cerebrovasculature during hypercapnia. We propose the extended cycles of hyperventilation associated with the HIIT protocol may have a prolonged alteration to the tone of the cerebral vessels causing them to remain slightly constricted following the exercise bout and thus the maximal dilatory capability of the MCA and PCA would have been compromised, consistent with previous research in this area (Marsden et al., 2012; Ogoh & Ainslie, 2009b; Ogoh et al., 2005). Conversely, although the MICT session likely resulted in the cerebral vessels being vasodilated during the exercise intervention, this only had a minor impact, exclusively attenuating the MCA relative hypercapnic slope at hours zero and one (Figure 4a). The rationale for this finding can be attributed to the cerebrovasculature tone being altered as a result of being vasodilated for a prolonged duration, and thus was unable to maximally dilate to carbon dioxide following MICT exercise. Moreover, the differential in findings of the severity of attenuation between exercise conditions may be attributable to rapid recovery in these measures immediately upon the cessation of moderate exercise, which were more pronounced following heavier exercise (Figures 3 and 4). This is supported within Table 1, as the CrCP was found to be more extensively impacted during and following the HIIT exercise, compared to the MICT, which only altered the CrCP during the exercise bout.

Moreover the alterations to the cerebral vessel tone in the current investigation appears to be isolated with respect to the hypercapnic vasodilatory response as the hypocapnic curves were comparable following all three conditions (Figures 3 and 4). This disparate finding across the physiologically range of P_ET_CO_2_ demonstrates the vasoconstriction associated with hypocapnic stimuli is likely unaltered following either HIIT or MICT. This is consistent with the idea the cerebrovascular tone is augmented, as the vasoconstrictive properties of these vessels appear to be intact to vasoconstrictive stimuli. The proposed alterations to cerebral tone following the HIIT protocol are likely very minor as there were no significant differences noted in absolute CBV at the associated P_ET_CO_2_ levels.

### 4.3 Within‐day reproducibility of cerebrovascular reactivity metrics

While several studies have examined variation of CVR measures within a 24‐hr timeframe, the findings from these are equivocal. Several investigations have reported a decrease in the CVR response to P_ET_CO_2_ alterations in the morning compared to the previous evening (Ainslie et al., 2007; Cummings et al., 2007; Meadows et al., 2005). Contrarily, another study reported an increased CVR response the morning after testing (Strohm et al., 2014). While the combined findings within these prior investigations demonstrate there are potentially alterations to CVR from a preceding evening to the next morning across days, the current investigation revealed there was little deviation in CVR measures *within* the same day. The variation in findings between the prior research and the current investigation may be partially due to the differences in experimental designs. Namely, the current study assessed CVR across the *same day* at several time points which enabled the head‐frame and TCD probes to remain locked in place while the previous investigations reapplied the TCD between trials occurring before and after sleep (Ainslie et al., 2007). Furthermore, the methodology in this study challenged the participants on both the hypocapnic and hypercapnic slopes across the functional physiologic carbon dioxide range, as they were measured from ranges of 25–40 mmHg and 40–60 mmHg, respectively (Figure 1). This ensured all participants reached maximal physiologically relevant ranges for vasodilation and vasoconstriction (Willie, Tzeng, Fisher, & Ainslie, 2014), allowing us to capture the complete picture of CVR parameters. The study most consistent with our current design was performed by Strohm and coworkers (2014) who assessed the day‐to‐day reproducibility of CVR measures across a P_ET_CO_2_ (range: 35–50 mmHg) and showed that although a circadian rhythm was present, the values associated with each testing time point were highly reproducible from one day to the next. This finding is consistent with the baseline measures in the current investigation and our results extend the notion that CVR metrics for a given time point in the day can be reproduced for testing sessions greater than three days apart (as indicated by the low within subject CoV values of <10%) (Figures 3 and 4).

Another possible explanation for the varying results associated with the reproducibility of CVR metrics may be attributed to the various methodologies used to examine these measures. For example, Meadows et al., (2005) used a face mask connected to a pneumotachograph and assessed people during five minutes of hypercapnia at a gas concentration of 79% carbon dioxide and 21% oxygen and flow rates from 0 to 800 ml/min (resulting in a P_ET_CO_2_ of 44.6 ± 1.3 mmHg). With this approach, they demonstrated a reduction to the hypercapnic cerebral vascular reactivity response in 14 of their 18 subjects the following morning (Meadows et al., 2005). Two of the previous studies had subjects breathe a fixed concentration of 5% carbon dioxide with 21% oxygen and a balance of nitrogen for three minutes following an eight‐minute baseline (absolute P_ET_CO_2_ levels not reported), finding a decrease as well (Ainslie et al., 2007; Cummings et al., 2007). Finally, Strohm and colleagues (2014) examined CVR of end tidal values ranging from 35 mmHg to 50 mmHg. These authors had participants breathe at eucapnia (40 mmHg) for two minutes before inducing hypercapnia (50 mmHg) for five minutes. This was followed by a slow decline to hypocapnia (35 mmHg), which was held for three minutes before returning to hypercapnia (50 mmHg) and then eventually eucapnia. Therefore, the majority of previous research examining within‐day variation associated with CVR has only investigated the response to hypercapnia, with just one previous investigation including a very mild hypocapnic stimulus and none extending their findings to the complete physiologically relevant range of P_ET_CO_2_ known to affect the cerebrovasculature (~25 mmHg to ~60 mmHg) (Willie et al., 2012). Thus, our study is the first to investigate the complete physiologically relevant range of P_ET_CO_2_ with respect to potential replicable measures and our data indicate CVR was not affected across the day, despite previous findings of circadian variation in heart rate (Conroy et al., 2005) and blood pressure (Degaute, Van De Borne, Linkowski, & Van Cauter, 1991; Kawano, 2011) measures. Based on our results, we propose a ramp rebreathing method to index CVR results in highly reproducible values, which were comparable across all time points within this investigation (Figures 3 and 4).

### 4.4 Implications for future cerebrovascular reactivity assessments

The findings of this investigation will prove useful for researchers examining cerebrovascular parameters in the future for several reasons. First, these results enable researchers to give an objective time restriction related to the inclusion of participants who have recently exercised to ensure their results will not be skewed by acute exercise. This reduces the recommended 12 to 24 hr time restriction to two hours for assessing CVR metrics, which leads to increased ability to test active individuals or sporting teams who commonly have daily training sessions and/or games. Second, as this study examined different intensities of exercise, the results can be used to generalize the extent both steady state and short, maximal shift sports acutely alter the cerebrovasculature. Lastly, the results of this study allow for valid interpretability of CVR metrics across the typical workday as variation in CVR metrics in the control data has shown these effects to be negligible.

### 4.5 Limitations

A well‐known limitation of using TCD is its inability to measure flow and the assumption that velocity can be a surrogate measure for flow based on the supposition the diameter of the insonated vessel remains constant. Recently, two studies involving high resolution magnetic resonance imaging of the cerebral vessels (Coverdale, Gati, Opalevych, Perrotta, & Shoemaker, 2014; Verbree et al., 2014) have shown when P_ET_CO_2_ is within 8 mmHg of eucapnia the diameter of the cerebral arteries are relatively constant (Ainslie & Hoiland, 2014). While the current study employed P_ET_CO_2_ extending beyond this window, the interpretability of the results likely still holds true. For example, although the TCD will underestimate the alterations to flow associated with dilation in the cerebrovasculature, this likely only further highlights the discrepancies noted in the current investigation following HIIT, as the results are more likely to occur at an even greater extent if we were to assess a true measure of cerebral blood flow. However, future investigations with transcranial color‐coded duplex ultrasound will be able to confirm this speculation. Nevertheless, as all subjects performed all aspects of the investigation the authors believe the current findings provide strong and meaningful evidence exercise modalities do impact the CVR response and must be taken into account with the study design process. Another caveat is that the rebreathing protocol assumes CO_2_ production within the brain is similar to whole body CO_2_ production; however, this caveat likely had minimal impact on the current investigation as previous research has shown the body and brain have similar metabolism (Ainslie & Duffin, 2009). Furthermore, a limitation to the methodology utilized in this project, was the fact that hypercapnia was assessed prior to hypocapnia for each trial. Nonetheless, P_ET_CO_2_ was given sufficient time between the two assessments to return to eucapnic levels before transitioning to the hypocapnic trials, thus limiting the likelihood the first test would influence the second (Figure 1). Moreover each participants VO_2max_ was not individually determined, but rather HRR was predicted based upon the formula for maximal HR (220‐age). However, as all participants were at a similar self‐reported level of fitness (recreationally active) we are confident there was little impact on the cerebrovascular measures performed in the current investigation. Furthermore, it has been identified through a recent series of articles; cerebrovascular fitness levels only appear to alter cerebrovascular regulation in highly‐trained athletes (Favre & Serrador, 2019; Labrecque et al., 2017; Labrecque, Rahimaly, et al., 2019; Labrecque, Smirl, & Brassard, 2019b). While the respiratory exchange ratio was not quantified in the current investigation, the authors are confident each MICT was performed below anaerobic threshold. Additionally, a major limitation is the small sample size used within this investigation (*n* = 9). Nonetheless, given the extensive time requirements needed from each participant due to the nature of the cross‐over design, a smaller sample was used to ensure each individual could be their own control in each condition. Finally, all participants were all recreationally active young adults selected from a university setting, the findings may not be directly generalizable to clinical or elderly populations. These populations may require longer duration of recovery after exercise, before CVR measures can be collected and further research into these populations are warranted. Conversely, individuals with high to exceptional levels of fitness may have quicker recovery trajectories and thus, further research is needed understand the recovery‐time course with respect to cardiorespiratory fitness levels.

## 5 CONCLUSIONS

In summary, the findings from this investigation revealed CVR hypercapnic slopes were attenuated following the HIIT protocol in both the MCA and PCA. This alteration indicates there was a prolonged change to the tone of the main conduit vessels in the brain which did not return to baseline until hour two of exercise recovery. Moreover there were no changes found in any condition within the hypocapnic CVR slopes, further demonstrating the augmentation to the cerebrovascular tone limits the ability of the vessels to vasodilate in response to vasodilatory stimuli. Thus, studies wishing to employ cerebrovascular reactivity assessments should have participants abstain from exercise for at least two hours. Finally, CVR measures extending the entire range of the physiologically relevant P_ET_CO_2_ levels were not affected in the control aspect of the current investigation and therefore assessments performed at any time across the typical workday (8:00 a.m. to 7:00 p.m.) will yield valid and highly reliable results.

## CONFLICT OF INTEREST

None.

## AUTHOR CONTRIBUTIONS

J.S.B., K.J.B., and J.D.S. designed the study. J.S.B., P.C., A.M., O.M., and J.D.S. performed data collections. J.S.B. and J.D.S. performed statistical analysis and interpreted the data. J.S.B., P.C., A.M., O.M., K.J.B., and J.D.S. contributed to writing and proofing the final version of the manuscript.
